# PReS-FINAL-2234: Successful canakunimab treatment in uveitis secondary to cryopyrin-associated periodic syndrome

**DOI:** 10.1186/1546-0096-11-S2-P224

**Published:** 2013-12-05

**Authors:** O Kasapcopur, I Tugal-Tutkun, K Barut, T Erener-Ercan, A Gul

**Affiliations:** 1 Pediatric Rheumatology, Istanbul University, Cerrahpasa Medical Faculty, Istanbul, Turkey; 2 Ophthalmaology, Istanbul University, Istanbul Medical Faculty, Istanbul, Turkey; 3 Rheumatology, Istanbul University, Istanbul Medical Faculty, Istanbul, Turkey

## Introduction

Cryopyrin-associated periodic syndrome (CAPS) is a constellation of diseases with a different and varying clinical spectrum which is rarely seen in childhood. The mildest form of CAPS which is familial auto-inflammatory syndrome and the most severe form which is chronic inflammatory neurologic cutaneous arthropathy (CINCA) possess a very different spectrum of clinical findings. Most important organ involvement in CAPS is the associated uveitis. Uveitis in CAPS is unresponsive to most of the classic treatment modalities.

## Objectives

In this case report, we will describe the response of a patient to canakunimab therapy whose uveitis associated with CAPS was resistant to other traditional treatment modalities.

## Methods

### Case report

The female patient who was healthy until 3 years of age developed urticarial rash associated with a 4 to 5 day duration of fever which started to occur periodically every 1 month. At 5 years of age, in addition to her periodic complaints of fever and rash, arthritis occurred in her left knee. After her admittance to our Rheumatology Department with these complaints, chronic arthritis of the right ankle was also detected. Her laboratory analysis revealed a normal complete blood count while acute phase reactant levels were elevated. ANA and RF were found to be negative in the patient. Ophthalmologic evaluation revealed episcleritis and chronic iridocyclitis. The patient was given methotrexate, prednisone and infliximab with varying doses and with varying durations. Despite different treatment modalities, her uveitis and urticarial findings persisted. NLRP3/CIAS1 mutation was investigated with the suspicion of CAPS. T436A mutation was detected. Neurosensorial hearing loss was not present in the patient. With the confirmation of diagnosis of CAPS, patient was given canakunimab therapy at a dose of 4 mg/every 2 months. After the first dose, joint and skin findings of the patient resolved completely. Her uveitis which was unresponsive to all of the treatment modalities also resolved after the first dose. Leukocytes in the vitreus also disappeared. The patient is now at the 18th month of canakunimab therapy and is totally asymptomatic without any flare of her uveitis.

## Conclusion

CAPS should be considered in the differential diagnosis in children presenting with an urticarial rash and oligoarthritis. If there is uveitis associated with CAPS, one of the most efficacious and safe drug for treatment is the anti-interleukin 1β agent, canakunimab.

## Disclosure of interest

O. Kasapcopur Consultant for: Novartis, I. Tugal-Tutkun: None declared, K. Barut: None declared, T. Erener-Ercan: None declared, A. Gul: None declared.

